# Hospitalisation and mortality outcomes after second COVID-19 vaccination among Australians living with severe mental illness: Findings from a matched case–control study using national data linkage

**DOI:** 10.1177/00048674261475206

**Published:** 2026-08-20

**Authors:** Claudia Bull, Amanda J. Wheeler, Victoria J. Palmer, Matthew Lewis, Dan Siskind, Steve Kisely

**Affiliations:** 1 The ALIVE National Centre for Mental Health Research Translation, The University of Queensland, Brisbane, Australia; 2 Queensland Centre for Mental Health Research, Faculty of Health, Medicine and Behavioural Sciences, The University of Queensland, Brisbane, Australia; 3 Princess Alexandra Hospital Southside Clinical Unit, Greater Brisbane Clinical School, Medical School, The University of Queensland, Brisbane, Australia; 4 The ALIVE National Centre for Mental Health Research Translation, Griffith University, Brisbane, QLD, Australia; 5 Centre for Mental Health, Griffith University, Brisbane, QLD, Australia; 6 Faculty of Medical and Health Sciences, Auckland University, New Zealand; 7 The ALIVE National Centre for Mental Health Research Translation, The University of Melbourne, VIC, Australia; 8 The Department of General Practice and Primary Care, Faculty of Medicine, Dentistry and Health Sciences, The University of Melbourne, VIC, Australia; 9 Metro South Addiction and Mental Health Service, Metro South Health, Brisbane, QLD, Australia

**Keywords:** COVID-19, vaccination, severe mental illness, hospitalisation, mortality

## Abstract

**Objective:**

To compare the proportion of Australians living with severe mental illness (target group) and the general population (comparator group) who received the first, second, and third doses of the COVID-19 vaccination between 22 February 2021 and 30 June 2022; examine the odds of any, non-psychiatric, and COVID-19-related hospitalisations within 12 months of the second vaccination; and examine the odds of all-cause and COVID-19-specific mortality within 12 months.

**Methods:**

We used a retrospective 1:4 age- and sex-matched case–control design. Subgroup analysis of individuals aged ⩾65 years old was also undertaken. This study used de-identified, linked administrative data from the Australian Immunisation Register, Pharmaceutical Benefits Scheme, Medicare Benefits Schedule, National Death Index, Demography data, and hospital records.

**Results:**

The cohort included 375,335 individuals living with severe mental illness and 1,373,231 comparators. Between 22 February 2021 and 30 June 2022, the proportion of target group members receiving the first, second, and third doses was significantly lower than the comparators, albeit still high, and with a shorter average time between vaccinations. Adjusted odds of hospitalisation in the target group within 12 months of the second vaccination were 14.0 (95% CI = 13.8–14.2) for any, 9.7 (95% CI = 9.6–9.8) for non-psychiatric, and 19.0 (95% CI = 17.7–20.3) for COVID-19-related hospitalisations. Adjusted odds of all-cause mortality were 3.1 (95% CI = 3.0–3.1) and 9.4 (95% CI = 7.7–11.5) for COVID-19-related mortality.

**Conclusions:**

People with severe mental illness had significantly poorer hospitalisation and mortality outcomes after their second vaccination, highlighting persistent gaps in access and equity of care for this vulnerable population.

**Study registration:**

ACTRN12624000117516.

## Introduction

At the beginning of the COVID-19 pandemic, individuals living with severe mental illnesses such as schizophrenia were identified as a high-risk population for contracting the virus (Fond et al., 2021; Vai et al., 2021; Wang et al., 2021). This is because they often have unmet physical health needs, and reduced access to medical care, complicated by environmental and lifestyle factors (Fond et al., 2021; Vai et al., 2021; Wang et al., 2021). Thus, there is a clear ethical imperative to ensure vaccination equity (i.e. fair access to and uptake of vaccinations; United Nations Development Programme, 2021) for individuals living with severe mental illnesses (De Hert et al., 2021; Warren et al., 2021).

Australia’s COVID-19 vaccination programme commenced on 22 February 2021 (Department of Health and Aged Care, 2021). However, 5 months into the vaccine rollout, only 11.8% of Australia’s population had received both vaccinations compared with over 50% of people in Canada (50.2%), the United Kingdom (UK, 52.7%), Chile (62.0%), and Iceland (63.9%) (Mathieu et al., 2020). Aotearoa (New Zealand [NZ]) had a similarly slow start (12.5% of the population fully vaccinated in the first 5 months; Mathieu et al., 2020). Since entering a post-pandemic era, Australia’s response to COVID-19 has come under increased scrutiny with concerns that it exacerbated existing societal inequalities for people of low socioeconomic status (SES), women, children, those in aged care, people with disabilities, temporary migrants, and people in multicultural communities (Grattan, 2022; Stobart and Duckett, 2022; Vally and Bennet, 2021). While Australia saw early success in limiting COVID-19 cases and deaths, by the middle of 2022 the COVID-19 death rate exceeded the Organisation for Economic Co-operation and Development average by 16% (Grattan, 2022).

To date, COVID-19 vaccination uptake in Australians living with severe mental illnesses remains unknown, and studies from other countries have reported mixed findings for this population. An NZ study revealed that only 61% of people with mental health issues, and 50% of people with substance use issues, had received their first COVID-19 vaccination by 29 September 2021, compared with 79% of the eligible population (Every-Palmer et al., 2022). This was reduced to 30% and 23%, respectively, for the second COVID-19 vaccination compared with 48% of the eligible population (Every-Palmer et al., 2022). A 2021 study from Israel reported that the vaccination rate in people with schizophrenia was 50.6% compared with 52.8% in the general population (Bitan et al., 2021). After adjusting for demographic and clinical factors, the hazard ratio (HR) for people with schizophrenia was 0.83 (95% CI = 0.81–0.86; Bitan et al., 2021). Six months later, 79.3% of people with schizophrenia had received their first vaccination compared with 85.5% of the general population (odds ratio [OR] = 0.65, 95% CI 0.62–0.67), although the proportion receiving two vaccinations was similar (Bitan et al., 2022). A case–control study from Ontario, Canada, showed that people with major mental illness were only slightly less likely to have their first (HR = 0.97, 95% CI = 0.96–0.98) and second (HR = 0.94, 95% CI = 0.93–0.95) vaccinations compared with the matched controls (matched for age, sex, and geography). Finally, research from the United States (US) involving approximately 4.9 million Veterans Health Affair (VHA) patients showed that individuals living with severe mental illnesses were equally as likely as the general population to receive a COVID-19 vaccination between 1 December 2020 and 1 June 2021 (48% and 46%, respectively; Haderlein et al., 2022).

Given that individuals living with severe mental illnesses are less likely to receive preventive or guideline appropriate healthcare for conditions such as cardiovascular disease and cancer (Bull et al., 2025; Kisely et al., 2023, 2024a, 2024b; Lorenz et al., 2013; Miles et al., 2020; Warren et al., 2021), we sought to compare the proportion of Australians living with severe mental illness (target group) and the general population (comparator group) who received the first, second, and third doses of COVID-19 vaccinations between 22 February 2021 and 30 June 2022. We also examined the odds of any, non-psychiatric, and COVID-19-related hospitalisations within 12 months of the second vaccination, as well as all-cause and COVID-19-specific mortality within 12 months.

## Methods

### Study design

This was a retrospective 1:4 age- and sex-matched case–control study using routinely collected, de-identified, linked administrative health data from the Australian Institute of Health and Welfare (AIHW) National Health Data Hub (NHDH). The study was prospectively registered as an observational study on the Australian New Zealand Clinical Trials Registry (ANZCTR, registration number: ACTRN12624000117516) and followed STROBE guidance (von Elm et al., 2008). We received ethical approval to undertake this study from the AIHW Human Research Ethics Committee (HREC, EO2022/3/1352), the University of Queensland HREC (2022/HE000416), and the Metro South HREC (HREC/2023/QMS/94167).

### Data sources

The NHDH is a major national health data linkage system for health research comprising de-identified data from state, territory, and Commonwealth data sources. For this study, we used data from Demography, Australian Immunisation Register (AIR), Pharmaceutical Benefits Scheme (PBS), Medicare Benefits Schedule (MBS), National Death Index (NDI), and admitted patient hospital datasets. Demography data contain personal project numbers unique to each individual in the dataset as well as their sex, First Nations status, birth month, and year. AIR is the national register of all vaccinations given to all Australians, including those provided under the National Immunisation Program, through school programmes, and privately administered (Services Australia, 2025). The PBS comprises data on all prescription medications subsidised by the Australian government, other than medications dispensed in-hospital (Department of Health and Aged Care, 2025). The MBS comprises claims data for all government (Medicare) subsidised health service utilisation by eligible Australians (Department of Health and Aged Care, 2024). The NDI is a listing of all deaths that have occurred in Australia since 1980, including the underlying cause of death (Australian Institute of Health and Welfare, 2023). Admitted patient hospital data are drawn from the National Hospital Morbidity Database, which compiles episode-level records from all Australian public and private hospitals except in Western Australia and the Northern Territory (Australian Institute of Health and Welfare, 2024b).

### Cohort derivation

Both the target and comparator groups included individuals who were aged ⩾18 years as of 22 February 2021, identified in the Demography data. The target group (i.e. individuals living with severe mental illnesses) were those who had been dispensed at least two prescriptions of authority-only antipsychotic medications used specifically for severe mental illnesses (Anatomical Therapeutic Chemical [ATC] classification codes N05AH, N05AX, N05AE, N05AX) within 12 months of each other, or two prescriptions for lithium within 12 months of each other, since 22 February 2019 (2 years prior to the period of interest of this study). In the case of quetiapine (ATC Code N05AH04), we only included individuals prescribed at least 100 mg on two occasions, to minimise the influence of off-label use. In Australia, atypical antipsychotic medications and lithium are the primary treatments for serious mental illness (SMI). PBS prescriptions for atypical antipsychotics necessitate a specific authority code indicating whether the condition being treated is schizophrenia or bipolar affective disorder while lithium is predominantly used for the latter, although it is also sometimes prescribed for treatment-resistant depression (Malhi et al., 2021). The use of lithium in other conditions such as neurodegenerative conditions and cluster headache is off-label or experimental (Lund et al., 2023; Singh et al., 2023).

The comparator group was matched on age and sex to the target group. That is, for every single member of the target group, four comparator group members were identified. Supplementary Material 1 clearly illustrates how the matched cohort was derived.

### Outcome measures

The outcomes of interest were the proportion of the population receiving the first, second, and third COVID-19 vaccinations; any, non-psychiatric, and COVID-19-related hospitalisations within 12 months of the second vaccination; and all-cause and COVID-19-related mortality within 12 months of the second vaccination. We used the second vaccination, rather than the first, given evidence of improved outcomes following two doses (Layton et al., 2025). Relevant COVID-19 vaccination codes were derived from AIR vaccination episode data (see Table 1). The sequence of vaccinations and time between vaccinations was determined using immunisation encounter dates. We selected a 12-month follow-up window to ensure that outcomes were temporally linked to the second vaccination while maintaining representativeness across the national rollout. Six months into the national rollout (22 August 2021), only 23.6% of the population had been vaccinated compared with 78% at 12 months (22 February 2022; Mathieu et al., 2020). Thus, restricting follow-up to a shorter window would have disproportionately captured early priority groups (e.g. healthcare workers, older people).

**Table 1.** COVID-19 vaccination codes and ICD-10-AM codes used for the classification of COVID-19.

COVID-19 vaccination codes (derived from AIR)	COMIRN, BHACOV, ASTCOV, MODERN, MODBIV, GAMSPU, COVAST, JANSSE, COMBIV, SINBBI, NOVNUV, MODBBA, COMBBA, MODPED, SINCOR
COVID-19 classification	ICD-10-AM codes in use
COVID-19 admission codes based on IHACPA guidance	
Coronavirus disease 2019 (COVID-19)	• U07.1 *Emergency use of U07.1*^a^ • U07.2 *Emergency use of U07.2*^a^ • U06.0 *Emergency use of U06.0*^a^ • U07.11 *Coronavirus disease 2019 [COVID-19], virus identified, asymptomatic*^b^ • U07.12 *Coronavirus disease 2019 [COVID-19], virus identified, symptomatic*^b^
Post COVID-19 conditions	• U07.3 *Emergency use of U07.3* • U07.4 *Emergency use of U07.4*
Multisystem inflammatory syndrome associated with COVID-19	• U07.5 *Emergency use of U07.5*
COVID-19 vaccines causing adverse effects in therapeutic use	• U07.7 *Emergency use of U07.7* • U07.70 *Coronavirus disease 2019 [COVID-19] vaccines, not elsewhere classified, causing adverse effect in therapeutic use* • U07.71 *Coronavirus disease 2019 [COVID-19] vaccine, using viral vector, causing adverse effect in therapeutic use* • U07.72 *Coronavirus disease 2019 [COVID-19] vaccine, using whole virus, causing adverse effect in therapeutic use* • U07.73 *Coronavirus disease 2019 [COVID-19] vaccine, using subunit, causing adverse effect in therapeutic use* • U07.74 *Coronavirus disease 2019 [COVID-19] vaccine, using nucleic acid, causing adverse effect in therapeutic use*
COVID-19 complicating pregnancy	• O98.5 *Other viral diseases in pregnancy, childbirth and the puerperium*
Additional COVID-19 admission codes based on Hitch et al. (2024)	
Lab-confirmed COVID-19 with symptoms, or Clinically diagnosed or probable COVID-19 with symptoms	• B97.2 *Coronavirus as the cause of diseases classified to other chapters to identify the infectious agent*
Lab-confirmed COVID-19 without symptoms, or Clinically diagnosed or probable COVID-19 without symptoms	• B34.2 *Coronavirus infection, unspecified site*
COVID-19 pneumonia, COVID-19 – pneumonitis, or Pneumonia – coronavirus disease 2019	• J128 *Other viral pneumonia* • J18.9 *Pneumonia, unspecified*

IHACPA: Independent Health and Aged Care Pricing Authority.

a Codes were deactivated from 1 July 2022.

b Codes were active from 1 July 2022.

COVID-19-related hospitalisations were determined using ICD-10-AM codes, and classified according to Independent Health and Aged Care Pricing Authority (IHACPA) guidance (see Table 1; Independent Health and Aged Care Pricing Authority, 2022). We also applied additional COVID-19 ICD-10-AM codes based on research noting that unspecified viral pneumonia and severe acute respiratory syndrome codes were initially applied to COVID-19-related admissions by health services before the World Health Organization (WHO) developed specific codes (Hitch et al., 2024). Non-psychiatric hospitalisations were identified using the mental health flag variable in the admitted patient data collection, specifically, those coded as non-ambulatory mental healthcare admission types. This analysis was performed to address potentially inflated admission rates among the target population.

Mortality within 12 months of the second vaccination included all-cause mortality and COVID-19-specific mortality. If an individual’s primary cause of death was coded U07.1, U07.2, U08.9, U09, or U10.9, this was considered a death due to COVID-19 as per the Australian Bureau of Statistics (ABS) coding schema (Australian Institute of Health and Welfare, 2024a). The second COVID-19 vaccination encounter date was used to determine the time between an individual’s second vaccination and their date of death.

### Covariates

Covariates included SES, geographical remoteness of residence, First Nations status, age, sex, and the presence of comorbidities. SES was based on the Socio-Economic Indexes for Areas (SEIFA), Index of Relative Socio-Economic Disadvantage (IRSD) as recorded in PBS data. This is an area-based (not individual-level) measure of socioeconomic disadvantage in quintiles from greatest disadvantage (quintile 1) to least disadvantage (quintile 5) (Australian Bureau of Statistics, 2021). The level of geographical remoteness was based on the Accessibility/Remoteness Index of Australia Plus (ARIA+) as recorded in PBS data. This measures an individual’s level of residential remoteness relative to their geographical access to services and is categorised according to Major Cities, Inner Regional, Outer Regional, Remote, and Very Remote Australia (Australian Bureau of Statistics, 2023). First Nations status was determined using admitted patient hospital data and indicates whether or not an individual identified as Aboriginal and/or Torres Strait Islander. Age and sex (as of 22 February 2021) were determined using Demography data. The presence of comorbidities was determined using PBS and MBS data. That is, if an individual had at least two MBS records indicating they were on a General Practitioner (GP) Management Plan, Team Care Arrangement, or GP Mental Health Treatment Plan within a 12-month period, or had at least two PBS records indicating the prescription of statins or hypoglycaemic agents within a 12-month period between 22 February 2019 and 22 February 2021, they were considered to have at least one comorbidity.

### Statistical analysis

After assessing data normality, records were removed if age was >100 years old and death records were ambiguous (i.e. an individual received a COVID-19 vaccination but was coded as dead before 22 February 2021). The sociodemographic characteristics of the target and comparator groups were described using frequencies, proportions, mean and standard deviation (SD), and median and interquartile range (IQR). All outcomes including proportion of people vaccinated; any, non-psychiatric, and COVID-19-related hospitalisations within 12 months of the second vaccination; and all-cause and COVID-19-related mortality within 12 months were similarly described. Differences between groups in terms of the average number of vaccinations received (COVID-19 vaccinations and all other vaccinations) were examined using independent sample t-tests with significance set at p < 0.05. Differences in the time between vaccinations one and two and two and three were examined using the Wilcoxon rank-sum test to account for non-normal data distribution.

Cox proportional hazards models were initially explored for hospitalisation and mortality outcomes. However, logistic regression was selected as the primary analytical approach because the study objective was to compare the occurrence of hospitalisation and mortality within a predefined 12-month period following the second vaccination. Firth’s bias correction was applied consistently across models to reduce potential small-sample bias in outcomes with lower event frequencies, particularly COVID-19-related mortality. Given the large sample size and high event counts for most outcomes, we would not expect the correction to have materially altered the substantive conclusions, but it provided a consistent analytical framework across all fitted models. Models were adjusted for SES, level of geographical remoteness, First Nations status, age, sex, and the presence of comorbidities as covariates. Although the target and comparator groups were matched on age and sex, analyses were adjusted for these covariates to account for differences in the risk of hospitalisation and mortality outcomes by age and sex. Unadjusted and adjusted logistic regression models using Firth’s bias correction were also used to estimate the odds of hospitalisation and mortality outcomes within 12 months in the subgroup of individuals aged ⩾65 years of age. All analyses were undertaken in SAS 9.4 (SAS Institute Inc.).

## Results

There were 375,335 target group members and 1,373,231 comparator group members. Table 2 shows the sociodemographic profile of the two groups. The average age of the target group was 51.1 ± 19.3 and 49.5 ± 18.3 for the comparator group. The proportion of individuals identifying as First Nations was markedly higher in the target group (8.2%) compared with the comparator group (2.9%), as was the proportion of individuals born in Oceania and Antarctica (80.9% vs 75.1%), in the SES quintile of greatest disadvantage (21.4% vs 16.4%), and with comorbid health conditions (77.3% vs 44.6%).

**Table 2.** Sociodemographic profile of the target and comparator groups.

Sociodemographic characteristic	Target group (n = 375,335)	Comparator group (n = 1,373,231)
Average age at first vaccination (SD)	51.1 (19.3)	49.5 (18.3)
Sex (n, %)		
Female	186,775 (49.8%)	673,906 (49.1%)
Male	188,560 (50.2%)	699,325 (50.9%)
First Nations status (n, %)^a^		
Yes	27,304 (8.2%)	30,787 (2.9%)
No	305,191 (91.8%)	1,030,894 (97.1%)
State/territory of residence (n, %)^b^		
ACT	5478 (1.5%)	22,752 (1.7%)
NSW	121,963 (32.5%)	423,870 (32.2%)
NT	2384 (0.6%)	10,594 (0.8%)
QLD	73,760 (19.6%)	256,329 (19.5%)
SA	28,852 (7.7%)	94,335 (7.2%)
TAS	8578 (2.3%)	29,162 (2.2%)
VIC	99,691 (26.6%)	338,780 (25.8%)
WA	34,443 (9.2%)	139,591 (10.6%)
Country of birth (n, %)^c^		
Oceania and Antarctica	241,032 (80.9%)	551,057 (75.1%)
North-West Europe	15,527 (5.2%)	49,703 (6.8%)
Southern and Eastern Europe	13,660 (4.6%)	28,429 (3.9%)
North Africa and the Middle East	6954 (2.3%)	16,767 (2.3%)
South-East Asia	6749 (2.2%)	25,451 (3.5%)
North-East Asia	3517 (1.2%)	18,653 (2.5%)
Southern and Central Asia	4734 (1.6%)	24,903 (3.4%)
Americas	2664 (0.9%)	8900 (1.2%)
Sub-Saharan Africa	3130 (1.1%)	9970 (1.3%)
Remoteness area (ARIA+) (n, %)^d^		
Major city	219,657 (59.9%)	815,023 (62.5%)
Inner regional	81,026 (22.1%)	271,217 (20.8%)
Outer regional	52,230 (14.2%)	167,819 (12.9%)
Remote	7629 (2.1%)	26,055 (2.0%)
Rural	6214 (1.7%)	22,896 (1.8%)
SEIFA Index of Relative Socio-Economics Disadvantage (IRSD) (n, %)^e^		
Quintile 1 (greatest disadvantage)	78,619 (21.4%)	213,768 (16.4%)
Quintile 2	71,548 (19.5%)	222,649 (17.1%)
Quintile 3	76,344 (20.8%)	265,892 (20.4%)
Quintile 4	67,153 (18.3%)	265,930 (20.4%)
Quintile 5 (least disadvantage)	72,967 (19.9%)	334,313 (25.7%)
Comorbidity (n, %)		
Yes	290,062 (77.3%)	611,802 (44.6%)
No	85,273 (22.7%)	761,429 (55.4%)

SD: Standard Deviation; ACT: Australian Capital Territory; NSW: New South Wales; NT: Northern Territory; QLD: Queensland; SA: South Australia; TAS: Tasmania; VIC: Victoria; WA: Western Australia; ARIA: Accessibility/Remoteness Index of Australia Plus; SEIFA: Socio-Economic Indexes for Areas; IRSD: Index of Relative Socio-economic Disadvantage.

a Missing data: 11.4% target group, 22.7% comparator group.

b Missing data: 0.1% target group, 4.2% comparator group.

c Missing data: 20.6% target group, 46.6% comparator group.

d Missing data: 2.3% target group, 5.1% comparator group.

e Missing data: 2.3% target group, 5.2% comparator group.

Table 3 shows that the proportion of target group members receiving the first (98.3%), second (96.0%), and third (68.9%) doses of the COVID-19 vaccination was significantly lower than in the comparator group (⩾99.0%, 98.1%, and 72.1%, respectively). Despite this, the proportion of both groups receiving their first and second vaccinations between 22 February 2021 and 30 June 2022 was high overall. Members of the target group had a significantly shorter time (in days) between receiving their first and second (median 28, IQR 21–68) and second and third (median 153, IQR 126–182) doses compared with the comparator group (median 31, IQR 21–76 and median 153, IQR 129–175, respectively). Figure 1 illustrates the proportion of target and comparator group members receiving the first, second, and third vaccinations across Australian jurisdictions. As the dose number increased, the variation between target and comparator group members receiving vaccinations increased across all jurisdictions.

**Table 3.** Proportion of the cohort vaccinated against COVID-19 between 22 February 2021 and 30 June 2022.

	Target group (n = 375,335)	Comparator group (n = 1,373,231)
Vaccination rates (n, %)		
⩾1 COVID-19 vaccination	369,007 (98.3%)	1,360,145 (99.1%)***
⩾2 COVID-19 vaccinations	360,341 (96.0%)	1,346,915 (98.1%)***
⩾3 COVID-19 vaccinations	258,493 (68.9%)	989,596 (72.1%)***
Average number of vaccinations received (mean, SD)		
Average number of COVID-19 vaccinations received	2.78 (0.79)	2.82 (0.70)***
Average number of other vaccinations^a^ received	3.80 (1.51)	3.89 (1.52)***
Average time (in days) between COVID-19 vaccinations (median, IQR)^b^		
Average time between COVID-19 vaccination 1 and 2	28 (21–68)***	31 (21–76)
Average time between COVID-19 vaccination 2 and 3	153 (126–182)***	153 (129–175)

SD: standard deviation; IQR: interquartile range.

a Other vaccinations include influenza, pneumococcal and diphtheria, tetanus, and pertussis vaccinations.

b Significance values estimated using Wilcoxon rank-sum test to account for non-normal data distribution.

* p < 0.05, **p < 0.01, ***p < 0.001.

**Figure 1.**
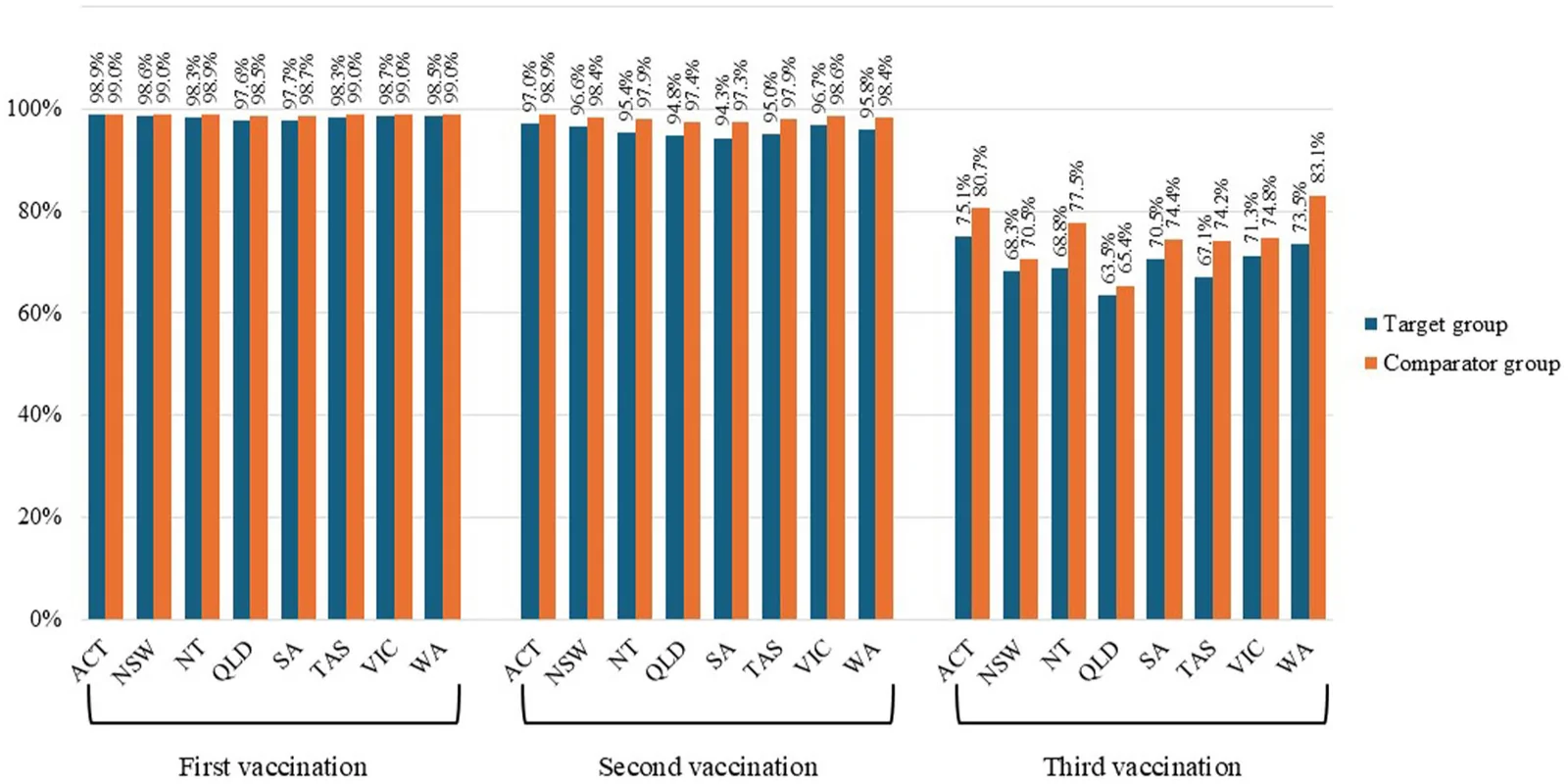
Differences in the proportion of target and comparator groups vaccinated by state and territory.

Table 4 shows that after model adjustment, the odds of the target group being hospitalised for any reason in the 12 months after their second vaccination were 14.0 (95% CI = 13.8–14.2) times higher than for the comparator group. In addition, their odds of non-psychiatric hospitalisations were 9.7 (95% CI = 9.6–9.8) times higher, and COVID-19-related hospitalisations were 19.0 (95% CI = 17.7–20.3) times higher than the comparator group. Subgroup analyses with individuals aged ⩾65 years of age revealed similar results of an even greater magnitude.

**Table 4.** Crude and adjusted odds of any, non-psychiatric, and COVID-19-related hospitalisations within 12 months of second vaccination^a^.

	Target group *n* (%)	Comparator group *n* (%)	Crude HR (95% CI)	Adjusted HR (95% CI)^b^
All hospitalisations	122,297 (32.6%)	43,384 (3.2%)	14.8 (14.6–15.0)***	14.0 (13.8–14.2)***
Non-psychiatric hospitalisations	94,346 (25.1%)	43,137 (3.1%)	10.3 (10.2–10.5)***	9.7 (9.6–9.8)***
COVID-19-related hospitalisations	6,267 (1.7%)	941 (0.07%)	24.7 (23.1–26.5)***	19.0 (17.7–20.3)***
Subgroup analysis, individuals aged ⩾65 years old	94,804 (25.3%)	304,881 (22.2%)		
All hospitalisations	38,295 (40.4%)	13,806 (4.5%)	14.3 (14.0–14.6)***	14.7 (14.4–15.0)***
Non-psychiatric hospitalisations	33,225 (35.0%)	13,714 (4.5%)	11.4 (11.2–11.7)***	11.5 (11.3–11.8)***
COVID-19-related hospitalisations	2767 (2.9%)	373 (0.1%)	24.5 (22.0–27.3)***	21.4 (19.2–23.8)***

OR: odds ratio; 95% CI: 95% confidence interval.

a Hospital data used in this table do not include data from Western Australia and the Northern Territory.

b Adjusted for SEIFA (Lowest quintile/greatest disadvantage vs all others), ARIA + (Major cities vs all others), First Nations status, age, sex, and comorbidity.

* p < 0.05, **p < 0.01, ***p < 0.001.

Table 5 shows that after model adjustment, the target group had 3.1 (95% CI = 3.0–3.1) times higher odds of all-cause mortality compared with the comparator group. Their odds of COVID-19-related mortality were also significantly higher at 9.4 (95% CI = 7.7–11.5). Subgroup analyses with individuals aged ⩾65 years of age revealed similar mortality results.

**Table 5.** Crude and adjusted odds of all-cause and COVID-19-specific mortality within 12 months of second vaccination.

	Target group *n* (%)	Comparator group *n* (%)	Crude OR (95% CI)	Adjusted OR (95% CI)^a^
All-cause mortality	21,774 (5.8%)	26,847 (2.0%)	3.1 (3.0–3.1)***	3.1 (3.0–3.1)***
COVID-19-related mortality	482 (0.1%)	130 (0.01%)	13.5 (11.2–16.4)***	9.4 (7.7–11.5)***
Subgroup analysis, individuals aged ⩾65 years old	94,804 (25.3%)	304,881 (22.2%)		
All-cause mortality	10,375 (10.9%)	7451 (2.4%)	4.9 (4.8–5.1)***	4.6 (4.4–4.7)***
COVID-19-related mortality	441 (0.5%)	117 (0.04%)	12.1 (9.9–14.9)***	9.7 (7.9–12.0)***

OR: odds ratio; 95% CI: 95% confidence interval.

a Adjusted for SEIFA (Lowest quintile/greatest disadvantage vs all others), ARIA+ (Major cities vs all others), First Nations status, age, sex, and comorbidity.

* p < 0.05, **p < 0.01, ***p < 0.001.

## Discussion

This is the first Australian study to compare the COVID-19 vaccination and subsequent hospitalisation and mortality outcomes between individuals living with severe mental illness and an age- and sex-matched comparator group. While the proportion of target group members receiving the first, second, and third COVID-19 vaccinations was significantly lower than the comparator group, uptake remained high overall (over 96% for the first and second doses). However, the target group experienced significantly higher odds of hospitalisation and mortality within 12 months of their second vaccination. Notably, the target group experienced 14 times higher odds of any hospitalisation, nearly 10 times higher odds of non-psychiatric hospitalisation, and 19 times higher odds of COVID-19-related hospitalisation. Mortality differences were also marked, with over threefold higher odds of all-cause mortality and nearly 10 times higher odds of COVID-19-related mortality. Subgroup analyses for individuals aged ⩾65 years revealed even greater disparities, with odds of COVID-19-related hospitalisation exceeding 21 times and mortality nearly five times higher than comparators. These findings suggest that vaccination alone cannot offset the entrenched health inequities experienced by people with severe mental illness, including comorbid health conditions, socioeconomic disadvantage, and systemic barriers to care. Moreover, they raise important questions about why near-equivalent vaccination uptake was achieved through the COVID-19 vaccination programme, but similar participation has not been observed in other public health initiatives.

Despite Australia’s initial lag in its COVID-19 vaccination strategy, the near-vaccination equity (i.e. fair access to and uptake of vaccinations; United Nations Development Programme, 2021) evidenced in this study is considerably higher than in many other parts of the world (Bitan et al., 2022; Every-Palmer et al., 2022; Fond et al., 2023; Haderlein et al., 2022; Richard et al., 2024). Other Australian public health programmes related to cardiovascular health and cancer screening, which have historically failed to produce similar participation rates (Bull et al., 2025; Kisely et al., 2023, 2024a; Palmer et al., 2018), may therefore benefit from the strategies used in the COVID-19 vaccination rollout. These include transparent and consistent communication about vaccine benefits and safety, widespread distribution of vaccination sites (including in rural and remote areas), strong collaboration with local health authorities and organisations to tailor campaigns to diverse populations, and the use of real-time data to address gaps in vaccination rates promptly (Australian National Audit Office, 2022). These strategies likely collectively contributed to a campaign that ensured near-equal proportions of individuals living with severe mental illness and the general population received the first and second doses of the COVID-19 vaccination, and could be replicated in other national public health programmes.

Despite high overall levels of vaccination, our findings showed significantly poorer hospitalisation and mortality outcomes in the 12 months after the second vaccination for individuals in the target group. Our findings are directionally consistent with the systematic review by Dang et al. (2024), although our effect sizes were much more pronounced. Dang et al.’s review found increased odds of COVID-19-related hospitalisation (pooled OR = 2.66, 95% CI = 1.13–6.22) and mortality (pooled OR = 2.67, 95% CI = 0.27–26.45) among vaccinated individuals with severe mental illness compared with the controls. However, the review also reported substantial between-study heterogeneity, a limited number of contributing studies, and relatively small numbers of COVID-related hospitalisation and mortality events. Accordingly, Dang et al.’s pooled estimates are not definitive benchmarks against which all subsequent studies are expected to align. Differences in cohort ascertainment, outcome definitions, follow-up periods, healthcare systems, and vaccine rollout strategies may contribute to variation in effect estimates across studies. Nonetheless, combined with our findings, there is evidence to suggest that individuals living with severe mental illnesses may be at a greater risk of contracting COVID-19 and experiencing poorer outcomes, despite being vaccinated. Reasons for this include increased exposure to the virus (Sporinova et al., 2019), poorer provision of accessible health literacy materials to enable self-care (Shinn and Viron, 2020), limited opportunities to work from home due to low-skill or temporary employment (Stuart, 2006), higher likelihood of comorbidities (Casey, 2005; Hert et al., 2011; Novak et al., 2020), and potential psychotropic medication contraindications (Govind et al., 2021; Hert et al., 2011), among others. These challenges are further compounded by the complexity of self-managing health across multiple settings and providers. As the virus evolves and the effectiveness of vaccines possibly diminishes, these individuals are likely to be at an even greater risk of contracting COVID-19 (Dang et al., 2024). Thus, targeted protection measures for people living with severe mental illness must be established to minimise the risk of contracting the virus in the first instance. In addition, co-designed strategies to address broader physical health inequities are crucial. This includes improving access to preventive healthcare, enabling improved health literacy, and ensuring coordinated care across different health services.

### Strengths and limitations

This study has several strengths, including a large, national sample. This enabled us to produce a cohort that was age- and sex-matched between the target and comparator group members (1:4 match, respectively), supporting the generalisability of the findings. Moreover, the comprehensiveness of the data available for this study via the NHDH allowed adjustment for covariates that could influence the outcomes of interest.

However, our findings should also be interpreted in light of their limitations. First, we used PBS data rather than medical records to identify the target cohort, limiting our case definition to individuals prescribed authority-only antipsychotic medications or lithium. Other studies have typically included a more severely ill cohort that required inpatient or community treatment from specialist mental health services rather than the prescription of selected psychotropic agents (Lawrence et al., 2013). However, expanding our definition to include other conditions like major depression would have been challenging, given the widespread use of psychotropic medications such as antidepressants for other illnesses. Moreover, evidence shows that nearly 95% of Australians living with severe mental illness have been prescribed medication in the previous 4 weeks to support their mental health management (Morgan et al., 2012), and the PBS covers approximately 80% of prescription medicines, while private prescriptions (not subsidised by the PBS) account for less than 10% of all medications (Department of Health, Disability and Ageing, 2024).

Second, despite the availability of multiple data sources within the NHDH, there was a relatively high proportion of missing data for First Nations status and country of birth. Thus, these groups may not be well represented in the findings. Third, we broadly defined the presence of comorbidities for this analysis; thus, greater nuance about specific types of comorbidities may be needed in the future to support individuals to understand their unique risk of morbidity and mortality outcomes. Fourth, due to low cell counts, we were unable to examine the risk of specific types of COVID-19-related admissions such as ‘COVID-19 complicating pregnancy’ and ‘Multisystem inflammatory syndrome associated with COVID-19’. However, it is also positive that there were few of these types of admissions recorded.

Fifth, hospitalisation analyses did not include records from Western Australia or the Northern Territory because these data were not available within the NHDH at the time of analysis. Although the study still captured admissions from the majority of Australian jurisdictions, Western Australia represents a substantial proportion of Australia’s geographic area, and the Northern Territory has a distinctive population profile characterised by greater remoteness and a higher proportion of First Nations peoples. Consequently, it is unclear whether inclusion of hospitalisation data from these jurisdictions would have altered the magnitude of the observed associations.

Finally, it is important to note that our findings relate to vaccine uptake during the initial rollout period (approximately 18 months) rather than sustained immunity. We did not examine the timing of subsequent doses nor booster coverage, which may influence protection against severe outcomes. For instance, there was an insufficient follow-up period to allow an examination of the effect of a third vaccination on subsequent hospitalisation and mortality outcomes, despite evidence of better outcomes following three doses (Abu-Raddad et al., 2022; Lu et al., 2025). Future research should explore the impact of recency of vaccination and booster uptake on hospitalisation and mortality risk in this population.

## Conclusion

Our findings demonstrated that while COVID-19 vaccination coverage in the target group approached near-equity with the comparator group, this did not translate into equitable health outcomes. Despite high overall uptake (over 96% for the first and second doses), the target group experienced significantly poorer outcomes within 12 months of their second vaccination. These disparities persisted even among older adults, underscoring compounded vulnerability. The findings highlight that vaccination alone cannot overcome the entrenched health inequities that people living with severe mental illness face. Optimising the physical health of this population is crucial and requires targeted protection measures and co-designed strategies to address broader health inequities. Lessons from the COVID-19 vaccination rollout can also inform and enhance other Australian public health campaigns.

## Supplemental Material

sj-docx-1-anp-10.1177_00048674261475206 – Supplemental material for Hospitalisation and mortality outcomes after second COVID-19 vaccination among Australians living with severe mental illness: Findings from a matched case–control study using national data linkage Supplemental material, sj-docx-1-anp-10.1177_00048674261475206 for Hospitalisation and mortality outcomes after second COVID-19 vaccination among Australians living with severe mental illness: Findings from a matched case–control study using national data linkage by Claudia Bull, Amanda J. Wheeler, Victoria J. Palmer, Matthew Lewis, Dan Siskind and Steve Kisely in Australian & New Zealand Journal of Psychiatry

## Data Availability

Data cannot be shared as we do not have permission from the data custodians or ethics approval to do so.
