# Supplementary material for: Hospitalisation and mortality outcomes after second COVID-19 vaccination among Australians living with severe mental illness: Findings from a matched case–control study using national data linkage
Source: Aust N Z J Psychiatry. 2026 Aug 20;60(10):993–1004. doi: 10.1177/00048674261475206 (PMC13620350; doi:10.1177/00048674261475206)
Supplement: Supplementary file 1 [file sj-docx-1-anp-10.1177_00048674261475206.docx]

 **Supplementary material 1:** Flow diagram of the cohort derivation
